# The Natural Flavonoid Fisetin Inhibits Cellular Proliferation of Hepatic, Colorectal, and Pancreatic Cancer Cells through Modulation of Multiple Signaling Pathways

**DOI:** 10.1371/journal.pone.0169335

**Published:** 2017-01-04

**Authors:** Mаhmoud Youns, Wael Abdel Halim Hegazy

**Affiliations:** 1 Department of Functional Genome Analysis, German Cancer Research Center (DKFZ), Im Neuenheimer Feld 580, Heidelberg, Germany; 2 Department of Biochemistry and Molecular Biology, Faculty of Pharmacy, Helwan University, Cairo, Egypt; 3 Department of Microbiology and Immunology, Faculty of Pharmacy, Zagazig University, Al-Sharqia, Egypt; University of South Alabama Mitchell Cancer Institute, UNITED STATES

## Abstract

Digestive cancers are major causes of mortality and morbidity worldwide. Fisetin, a naturally occurring flavonoid, has been previously shown anti-proliferative, anti-cancer, neuroprotective, and antioxidant activities. In our study, the anti-tumor activities in addition to regulatory effects of fisetin on some cancer cell lines were investigated. Data presented here showed that fisetin induces growth inhibition, and apoptosis in hepatic (HepG-2), colorectal (Caco-2) and pancreatic (Suit-2) cancer cell lines. Gene expression results showed that 1307 genes were significantly regulated in their expression in hepatic and pancreatic cell lines. 350 genes were commonly up-regulated and 353 genes were commonly down-regulated. Additionally, 604 genes were oppositely expressed in both tumor cells. *CDK5* signaling, NRF2-mediated oxidative stress response, glucocorticoid signaling, and *ERK*/*MAPK* signaling were among most prominent signaling pathways modulating the growth inhibitory effects of fisetin on hepatic and pancreatic cancer cells. The present analysis showed, for the first time, that the anti-tumor effect of fisetin was mediated mainly through modulation of multiple signaling pathways and via activation of *CDKN1A*, *SEMA3E*, *GADD45B* and *GADD45A* and down-regulation of *TOP2A*, *KIF20A*, *CCNB2* and *CCNB1* genes.

## Introduction

Digestive cancers are major causes of cancer mortality and morbidity in the world [1]. Gastrointestinal cancers include malignancies arising in the esophagus, gallbladder, pancreas, liver and bile ducts, small intestine, stomach, colon and rectum. The incidence and mortality rates of these tumors differ significantly. Approximately, one-fifth of the cancer incidence and nearly one-fourth of the cancer related deaths in the US were due to gastrointestinal cancers. Colorectal and gastric cancers are the most common gastrointestinal cancers all over the world [2]. The 5-year survival is 90% and 63%, respectively, when these malignancies are detected early and at localized stage [2,3]. Pancreatic cancer is one of the most lethal human cancers, with almost identical incidence and mortality rates [4]. Early detection of gastrointestinal tumors may significantly reduce deaths, and thereby, identification of novel biomarkers for early detection is an urgent need [3,5].

Recently, attention has been focused on the use of natural products, especially dietary sources, and their semi-synthetic derivatives to overcome human diseases including tumors [6]. Previous reports suggested that consumption of natural products including vegetables and fruits is associated with decreased incidences of many chronic diseases, including tumors. Recently, a study reported that some bioactive compounds in plants can activate or suppress multiple signaling pathways through targeting small molecules in cancer cells indicating the massive impact that natural products may have [7,8].

Fisetin (3,7,3′,4′-tetrahydroxyflavone) is a polyphenol, naturally occurring flavonoid and abundantly found in fruits and vegetables such as apple, strawberry, grape, persimmon, cucumber, and onion [9]. Previously, fisetin has shown anti-proliferative, anticancer, neuroprotective, and antioxidant activities [10–12]. In recent years, Fisetin has been reported to inhibit cell proliferation, migration and invasion, and induce apoptosis in several cancer types, such as colon cancer [13], glioma cancer [14], lung cancer [15], nasopharyngeal carcinoma [16], prostate cancer [17], and bladder cancer [18] and cervical carcinoma[19]. It also inhibits micro-ophthalmia associated transcription factor (MITF) in melanoma cells and inhibits invasion of melanoma cells via modulation of the MAPK and NF-κB pathways [20–22]. Recently, it was reported that fisetin causes cell cycle arrest, apoptosis (caspase-dependent), and potentiate the anti-tumor effect of Chemotherapeutics in triple-negative breast cancer cells [23].

Here, the growth inhibitory effect of fisetin on human cancer cell lines representing three different tumor types, hepatic, colorectal and pancreatic tumors, was investigated. Our results showed that fisetin inhibited cellular growth and proliferation, induced apoptosis, through activation of caspases in these tumors. Furthermore, expression analysis results pointed out that fisetin growth inhibitory effect was modulated through multiple signaling pathways including *CDK5* signaling, NRF2-mediated oxidative stress response, glucocorticoid signaling, and *ERK*/*MAPK* signaling. The present analysis showed, for the first time, that the anti-tumor effect of fisetin was mediated mainly through activation of *CDKN1A*, *SEMA3E*, *GADD45B* and *GADD45A* and down-regulation of *TOP2A*, *KIF20A*, *CCNB2* and *CCNB1* genes.

The present analysis is aimed to be a starting point for generation of hypotheses on significantly regulated candidate genes and for a more detailed functional analysis of individual transcripts for the activity of fisetin in tumor cells.

## Materials and Methods

### Cell lines and materials

The human hepatocellular carcinoma (HepG-2), the colorectal carcinoma (CaCo-2) and the human resistant pancreatic cancer (Suit-2), cells were used in our study. Cells were cultures and treated as previously described [4,24–26]. Briefly, cells were maintained in DMEM (Invitrogen, Carlsbad, CA) medium, supplemented with penicillin, and streptomycin and fetal bovine serum (FCS) (Invitrogen, Carlsbad, CA). All cell lines were tested with Mycoplasma detection kits (Minerva Biolabs, Berlin, Germany) and shown to be free of mycoplasma as previously mentioned [26].

### Effect of fisetin on cellular viability as determined by SRB assay

In order to evaluate the growth inhibitory effect of fisetin on different cancer cell lines, the colorimetric assay SRB (Sulforhodamine B assay) was used as previously described [27,28]. Briefly, cells were cultured with or without gradually increasing doses of fisetin for 48 h. After medium aspiration, the cells were fixed, stained and incubated at room temperature for 30 min. After washing, tris-base solution (10 mM) was added to dissolve retained SRB dyes. Finally, absorbance was measured using a precision microplate reader (Molecular Devices, Sunnyvale, CA).

### Caspase-glo 3/7 assay

The effect of fisetin on apoptosis was investigated by caspase 3/7 activity assay as we previously described [24,28,29]. Cells were either treated with fisetin (2 × IC_50_; IC_50_ and ½ × IC_50_) or untreated (control). After 6 h treatment, caspase reagent was added to each well, mixed and incubated for 1h at room temperature. Luminescence was then measured and caspase activity was expressed as percentage of the untreated control.

### Quantitative determination of prostaglandin E2

In order to investigate the inhibitory effect of fisetin on prostaglandin E2 (PGE2) production, the PGE2 EIA Kit (Cayman Chemicals, Michigan, USA) was utilized as we previously described [4,30]. Briefly, twenty four hours after fisetin treatment, cell lines were stimulated with arachidonic acid and prostaglandin E2 levels were estimated by a competitive enzyme immunoassay relative to untreated control.

### Expression profiling using array techniques

Cells were either treated with 10 μM fisetin or with DMSO (control) for 48 hours. RNA extraction, purification and microarray processing has been carried out as we described previously [4,24,26,29].

### Quantitative Real-Time RT-PCR Assays

The same RNA samples used for expression analysis experiment were used for RT-PCR (quantitative real-time) experiment for independent verification of the expression differences on some transcripts selected during analysis (e.g. *Top2A*, *CDKN1A*, *GADD45B*, *GADD45A* and, *CCNB2* and SEMA3E) with QuantiTect SYBR Green Kit (Qiagen) as previously described [24,29,31].

### Identification of networks and signaling pathways

Here, Ingenuity Pathway Analysis software (IPA) (Mountain View, USA) was used to identify list of networks of genes and canonical pathways modulated due to fisetin treatment of liver and pancreatic cancer cell lines (www.ingenuity.com). Using Ingenuity Pathway Analysis tool, significantly regulated transcripts were grouped and classified based on their biological importance and pathways involved in their effects.

### Data analysis

Microarray optimization, normalization and cluster analysis were performed as described in our previous studies [24–26,29]. Briefly, microarray quality assessment, correspondence cluster analysis and normalization were performed with the MIAME compatible analysis. Only variations with a p value of less than 5% were taken into account. Cluster analysis was performed using correspondence analysis [24–26,29,31].

### Cytochrome P450 (CYP3A4) inhibition assay

In order to monitor the inhibitory effect of fisetin on recombinant human CYP3A4, CYP450-Glo^TM^ assay (Promega, Mannheim, Germany) was used according to manufacturer instructions [32]. At room temperature, equal volumes of different compound concentrations and a reaction mixture containing CYP3A4 specific substrate (luciferin 6-benzyl ether in phosphate buffer) and CYP3A4 were incubated for ~10 min. The enzyme reaction was initiated after addition of NADPH regeneration system in citrate buffer. Thirty minutes later, 50μl luciferin was added. After 20 min, luminescence was recorded using a Tecan^TM^Safire II reader. Each experiment has been done at least 3 times (6 replica each). Ketoconazole was used as a positive control.

### Inhibition of Glutathione-S-transferase (GST) assay

In order to evaluate the possible inhibitory effect of fisetin on Glutathione-S-transferase enzyme, GST enzyme activity assay has been carried out. The method has been done as described previously [33]. Using 1-chloro- 2,4-dinitrobenzene (CDNB) as GST substrate. Briefly, untreated and treated cell lysates were used for this assay by preparing a sample with a standard assay mixture containing CDNB, reduced glutathione (GSH) and PBS buffer. The reaction was detected spectrophotometry at 340 nm.

## Results

### Fisetin inhibits cellular proliferation and viability of hepatic, colorectal and pancreatic cancer cell lines

**Fig 1:**
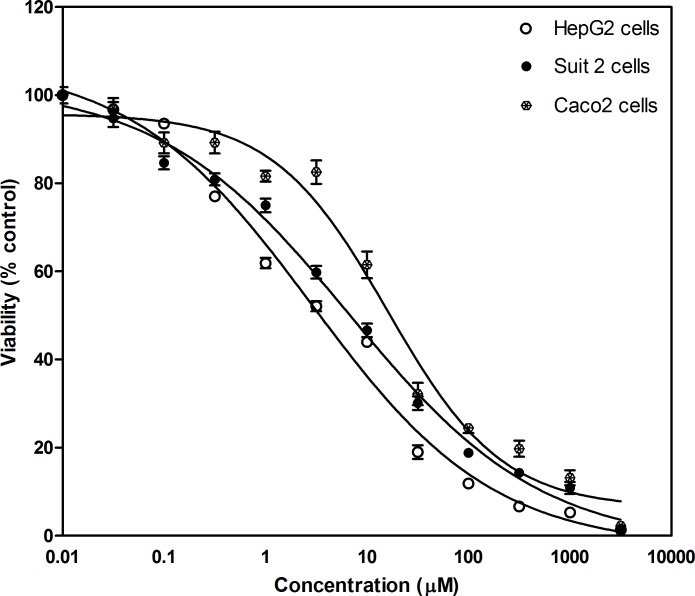
Fsitein inhibits growth of HepG-2, Caco-2 and Suit-2 cells. Exponentially growing cells were cultured and treated with fisetin for 2 days. The SRB assay was then performed to assess cell viability and proliferation. Results are represented as mean value (M) ± S.E.M. of at least 3 independent experiments (8 replica each).

In order to investigate the cellular anti-proliferative effect of fisetin on different cancer cell lines, HepG-2, Caco-2 and Suit-2 cells were cultured and treated with increasing concentrations of fisetin for 48h. The rate of cellular viability and growth was assessed using SRB assay. Fisetin showed growth inhibitory effects in a concentration dependent manner (Fig 1). The human liver cancer cells (HepG-2, IC_50_: 3.2μM) were more sensitive to fisetin effect compared to colorectal (Caco-2, IC_50_: 16.4 μM) and pancreatic cancer cells (Suit-2, IC_50_: 8.1μM).

### Apoptotic induction by fisetin is dose-dependent

**Fig 2:**
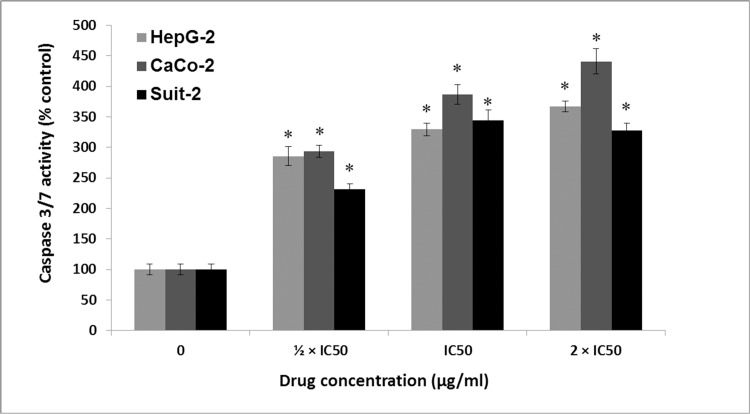
Apoptotic effect of fisetin as shown by measuring caspase 3/7 activatioin. Cells were treated with gradient concentrations of fisetin and caspase 3/7 assay was carried out. Results are presented as mean value (M) ± S.E.M. of at least 3 independent experiments (4 replica each), * significantly different from control group (P< 0.001) using 2 way ANOVA test.

Induction of apoptotic cascade is one of the central mechanisms of chemotherapy-induced cell death [34]. Determining whether the chemo-preventative effect of fisetin demonstrated above was a result of its ability to activate the apoptotic pathway; HepG-2, Caco-2 and Suit-2 cells were treated with or without fisetin for 6 hours then the activity of caspase 3/7 was measured using the Caspase-Glo 3/7 assay kit. Fisetin caused significant increase in activation of caspase 3/7 compared to untreated control and we concluded that fisetin induced apoptosis was mediated through activation of apoptotic cascade (Fig 2).

### Fisetin inhibits PGE2 production

**Fig 3:**
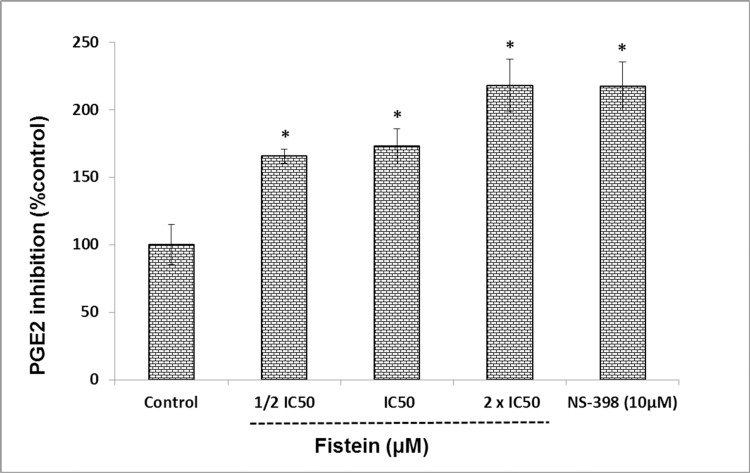
Inhibition of prostaglandin E2 after fisetin treatment of Caco-2 cells. Results are represented as mean value (M) ± S.E.M of at least three independent experiments (4 replica each). * Significantly different from control group (P< 0. 05) using one way ANOVA test.

Here the colorectal cancer cell line Caco-2 with higher COX-2 expression was used for the assay [29]. In order to evaluate the anti-inflammatory effect of fisetin through inhibition of PGE2 production, the PGE2 levels produced by cancer cells after fisetin treatment were determined. Our results showed that fisetin significantly inhibited PGE2 production in a dose-dependent manner (Fig 3).

### Identification of target molecules and pathways mediating the anti-proliferative effect of fisetin on cancer cells

**Fig 4:**
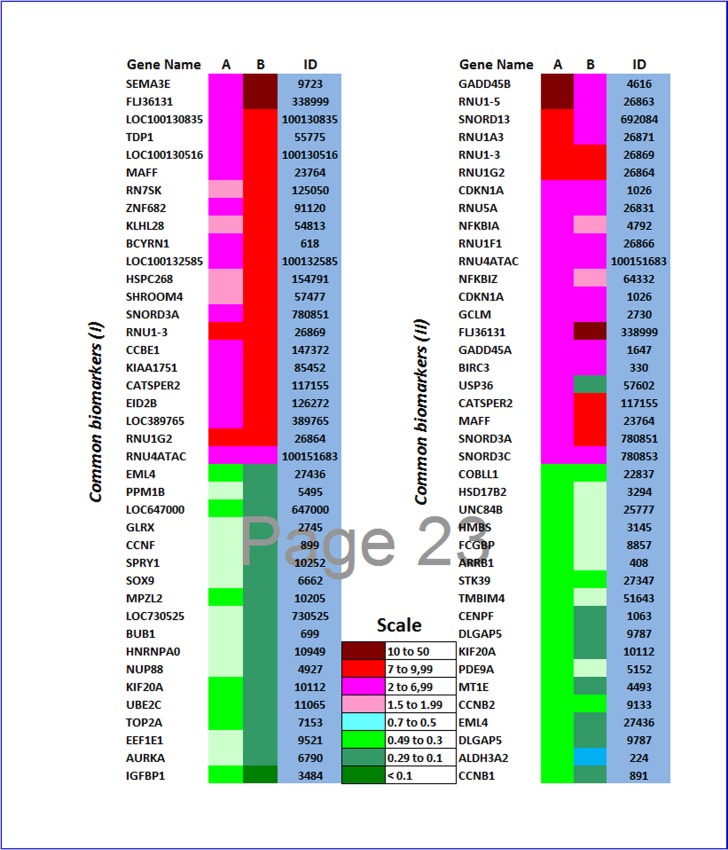
**Top 40 common biomarker genes expressed in both Suit-2 (A) and HepG-2 (B) cells.** Arranged in a descending order according to gene expression levels in Suit-2 (I) and HepG-2 (II), respectively.

For the identification of molecular targets, pathways and networks involved in the cytotoxic effect of fisetin, HepG-2, and Suit-2 cells were treated with or without 10 μM fisetin for 48h. Gene expression analysis has been carried out as previously described [24,26,29]. Microarray analysis results showed that fisetin commonly regulated 1307 genes in both HepG-2 and Suit-2 cells. 350 genes were commonly up-regulated and 353 genes were commonly down-regulated in both HepG-2 and Suit-2 cell lines. Additionally, 604 genes were oppositely expressed in both tumor cells. Regulated genes were mainly those involved in cell cycle, apoptosis, angiogenesis, and metestasis. The top 40 commonly up- and down-regulated in both cell lines were represented according to expression level in Suit-2 and HepG-2 cells, respectively (Fig 4.).

### Rt-PCR validation of expression analysis results

Expression of some significantly regulated genes after data analysis was then confirmed by RT-PCR, and normalized to GAPDH. Array results and Rt-PCR results were significantly matched (Table 1).

**Table 1:** Confirmation of microarray gene expression results with Rt-PCR.

*qRT-PCR verification of Microarrays results*			
Official gene name	Gene Symbol	Fold change (Microarray)	Fold change (qRT-PCR)
Topoisomerase (DNA) II alpha	*TOP2Aa*	- 2,17	- 1,87
Cyclin Dependent Kinase Inhibitor 1A	*CDKN1A*	+ 6,65	+ 4,75
Growth Arrest And DNA Damage Inducible Alpha	*GADD45A*	+ 4,14	+ 5,50
Growth Arrest And DNA Damage Inducible Beta	*GADD45B*	+ 13,03	+ 8,95
Cyclin B2	*CCNB2*	- 2,94	- 3,45
Semaphorin 3E	*SEMA3E*	+ 2,53	+3,90

### Pathway analysis and classification of significantly regulated genes

**Fig 5:**
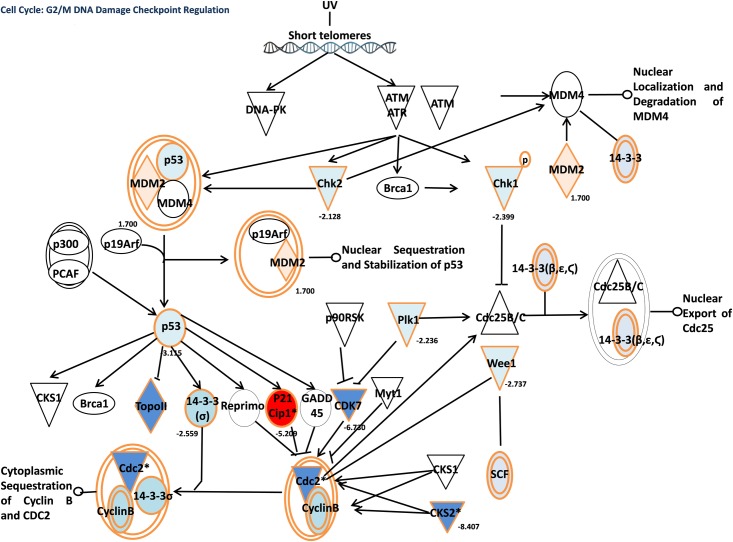
Cell cycle: G2/M DNA damage checkpoint regulation after treating HepG-2 cells with fisetin.

**Fig 6:**
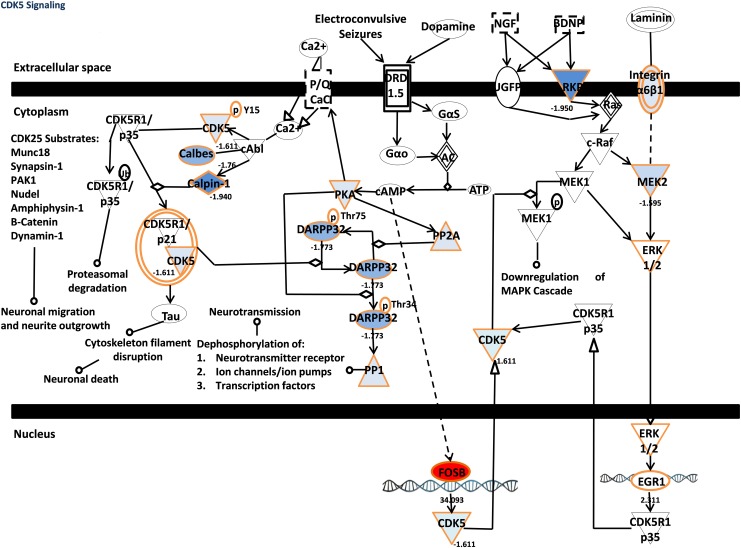
CDK5 signaling pathway after treating Suit-2 cells with fisetin.

In order to get deeper insights into canonical pathways and common mechanisms involved in the growth inhibitory effect of fisetin on hepatic and pancreatic cancer cells growth, the Ingenuity Pathway Analysis (IPA) tool was used [24]. The top five canonical pathways, regulated after fisetin treatment of HepG-2 cells were the mitotic roles of polo-like kinase, estrogen receptor signaling, protein ubiquitination pathway, cell cycle regulation (Fig 5.) and *NRF2*-mediated oxidative stress response. Additionally, the top five canonical pathways, regulated after fisetin treatment of Suit-2 cells were the *CDK5* signaling (Fig 6.), *ERK*/*MAPK* signaling, 4-1BB signaling in T-Lymphocytes, *NRF2*-mediated oxidative stress response, and *PI3K*/*AKT* signaling.

Moreover, the top molecular and cellular functions involved in the anti-proliferative effect of fisetin on HepG-2 cells were cell cycle, cell death and survival, protein synthesis, gene expression, and cellular growth and proliferation, while those for the effect of fisetin on Suit-2 cells were cellular growth and proliferation, cell death and survival, cellular development, cell cycle and cellular movement.

Biomarker analysis results showed that 1307 genes were significantly regulated in both cell lines after fisetin treatment. Common biomarkers significantly up-regulated in both cell lines included 350 genes, while 353 were commonly down-regulated in both cell lines indicating a common set of genes modulating the antitumor effect of fisetin. It was obvious that the NRF2-mediated oxidative stress response was specifically regulated after fisetin treatment of both tumor types. Among genes involved in NRF2 pathway and were significantly altered in their expression in our study are *NRF2*, *JUN*, *SOD*, *MRP4*, *ASK1*, *PI3K*, *FRA1*, and *PMA1* genes (data not shown).

### Effect of fisetin on cellular metabolism (CYP3A4 assay)

**Fig 7:**
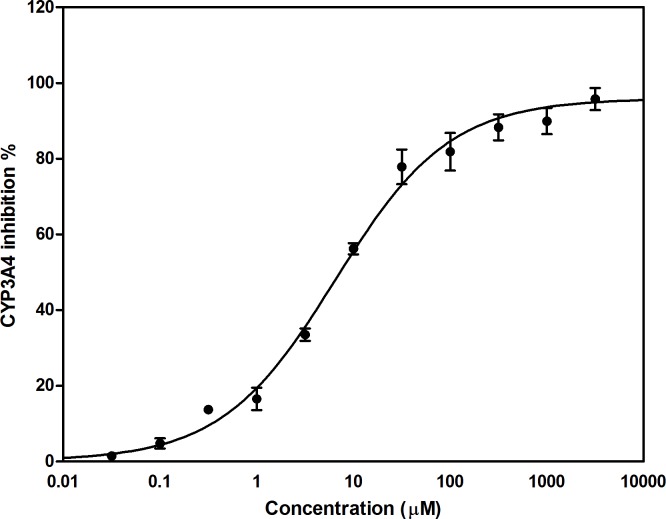
Effect of fisetin on CYP3A4 enzyme activity. Fisetin inhibits CYP3A4 activity in a dose dependent manner. Results are presented as mean value (M) ± S.E.M. of at least 3 independent experiments (6 replica each).

To estimate the inhibitory effect of fisetin on CYP3A4, CYP450-GloTM assay was carried out (Fig 7). The assay showed that fisetin caused a significant inhibition of CYP3A4 in a dose dependent manner and we concluded that fisetin induced cell death was, in part, due to its effect on cellular metabolism.

### Effect of fisetin on Glutathione-S-transferase enzyme (GST assay)

**Fig 8:**
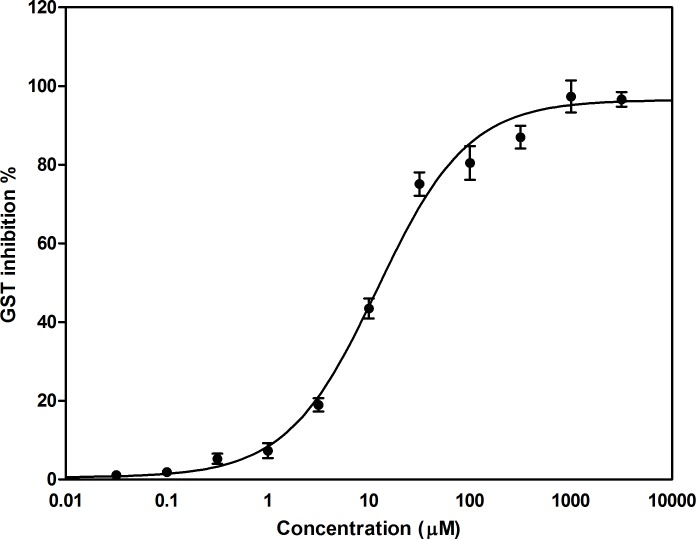
Effect of fisetin on GST enzyme activity. Fisetin inhibits GST activity in a dose dependent manner. Results are presented as mean value (M) ± S.E.M. of at least 3 independent experiments (6 replicas each).

In order to evaluate the probable inhibitory effect of fisetin on Glutathione-S-transferase enzyme, GST enzyme activity assay has been carried out. The results showed that fisetin induced enzyme inhibition in a dose dependent manner (Fig 8).

## Discussion

The deleterious side effects of present clinically used anticancer agents augment cancer mortality and morbidity and underscore an urgent need for new and safer remedies.

Dietary alterations can lead to widespread differences in the risks and incidences of several types of cancers. Additionally, the long term consumption of natural products present in fruits and spices, with proven safety, favoring their use in cancer chemoprevention [35]. The approach of tumor prevention using safe and nontoxic novel plant derived agents has been fortified by many scientists. To date, natural products and their synthetic or semisynthetic derivatives comprising huge number of approved anticancer drug candidates [24,36,37].

Plentiful natural products have been investigated for their potential use as anticancer agents. Among these agents, fisetin is a highly promising drug candidate. Fisetin is a bioactive flavonol molecule found in fruits and vegetables such as apple, grape, persimmon, strawberry, onion and cucumber [6].

Previous results showed that fisetin exhibits broad activity, including neurotrophic, anti-angiogenic, neuroprotective, radioprotective [15,38], and antioxidant effects [39]. In addition, several studies show that fisetin protects against several cancer types, including colorectal, prostate, breast, cervical, bladder, lung, and breast cancer [38,40,41]. Moreover, other reports showed that fisetin induces cell cycle arrest, apoptosis and suppress the growth of human colon cancer cells by inhibiting Wnt/EGFR/NF-kB and COX-2 signaling pathways [42]. Suppression of androgen receptor signaling in addition to apoptotic induction and cell cycle arrest in hepatocellular and prostatic carcinoma cells via the induction of p53 and p21(Waf/Cip-1) were also reported after fisetin treatment [43–45]. Fisetin induces autophagy through affecting TORC1 and mTORC2 pathways [46]. These results suggest that fisetin may be a useful anticancer or chemotherapeutic agent [15].

In spite of great efforts that have been previously done, the exact mode of action of fisetin on tumor cell growth is still unknown and needs to be further clarified. Here, the growth inhibitory effect of fisetin on three cell lines representing different tumor types namely hepatic, colorectal and pancreatic were investigated. Cellular viability was monitored by SRB cell proliferation assay. Apoptotic induction was investigated by measuring the amount of caspase 3/7 produced after treatment. Effect of fisetin on metabolic enzymes (Cytochrome P450 3A4 and Glutathione-S- transferase) and prostaglandin production (PGE2) was also evaluated.

Additionally, a transcript expression profiling study was carried out using microarray analysis technique to point out global genes, molecular targets, pathways that mediate the effect of fisetin on hepatic (HepG-2) and pancreatic cancer cell lines (Suit-2).

Our study showed that fisetin inhibited cellular growth and proliferation and induced apoptosis of all studied cell lines. Inhibition of cellular growth was more prominent with HepG-2 cells while the apoptotic effect was more prominent with Caco-2 cells. Fisetin also was able to inhibit cyctochrome P450 (CYP450 3A4) and glutatihione -S-transferase activity and the effect was dose dependent. Moreover, the anti-inflammatory effect of fisetin was confirmed through inhibition of PGE2 production in Caco-2 cells.

It was reported that a molecule, that is able to target multiple signaling pathways in cancer cells, could be a drug of choice to overcome multiple mechanisms and signaling pathways used by tumor cells to escape various defense mechanisms [47]. Here, using expression analysis technique, we proved that fisetin inhibited cellular growth through affecting multiple signaling pathways proposing its valuable role as safe antitumor agent.

Among the top 40 significantly up-regulated genes after fisetin treatment of both hepatic and pancreatic cancer cell lines is Axon guidance protein Semaphorin 3E (*SEMA3E*). It was previously reported that semaphorins (family of conserved membrane associated proteins) are secreted and use plexin proteins as their primary receptors for signal transduction. In cancers, de-regulation of semaphorins and their receptors is commonly observed [48]. Recent studies showed that *SEMA3E* expression is inversely correlated with tumor prognosis in pancreatic and colorectal cancer [49,50]. *SEMA3E* was among the common biomarkers significantly up-regulated in their expression after treatment of both cell lines with fisetin that may explain in part the growth inhibitory effect of fisetin on tumor growth.

The growth arrest and DNA damage inducible genes (*GADD45*) are principal players that play an important role in suppressing multistep carcinogenesis. They are mainly up-regulated during cellular stress. GADD45 plays an important role in suppressing multistep carcinogenesis. Over-expression of *GADD45* results in several processes of growth arrest, survival, apoptosis, or DNA repair [51]. Cell cycle arrest induced by *GADD45* was reported to be through interaction with *PCNA*, *P21*, and cyclin *B1* [52–54]. In addition to be involved in G1 or G2 cell cycle arrest, *GADD45B* has been implicated in and has been linked to the progression of HCC [55,56]. The fact that *GADD45B* and *GADD45A* were significantly overexpressed in both cell lines after fisetin treatment could explain that induction of cell cycle arrest was among mechanisms modulating the anti-tumor activity of fisetin on cancer cell lines.

Additionally, our gene exression results reported significant down-regulation of CNB1 and CNB2 in both tumor types after fisetin treatment, confirming cell cycle arrest induced by fisetin through up-regulation of GADD45A and GADD45B shown above and as indicated by IPA analysis too. It is well known that cyclin B1 is involved in G2-to-M transition and cyclin B2 is a G2/mitotic-specific factor. They both play a key role in the S-to-G2/M phases [57].

Moreover, Array results showed that fisetin inhibited significantly *KIF20A* expression in both hepatic and pancreatic cancer cells. Kinesin, present in all eukaryotes, is a microtubule-dependent molecular motor protein family, plays several roles in cellular functions, including chromosome alignment, cytokinesis, and chromosome segregation [58]. *KIF20A* (kinesin family protein member) is involved in tumor growth and progression [59]. Recent study reported that *KIF20A* played an important role in anti-tumor activity of genistein, and introduced *KIF20A* as a potential target for drug intervention of gastric cancer [59]. It was also reported that microinjection of anti-*KIF20A* antibody caused multinucleation of Hela cells [60]. In addition, knockdown of *KIF20A* gene in pancreatic cancer cells (PDAC) by siRNAs, inhibited their growth [61], implicating a vital role in cytokinesis and maintaining cellular viability. Here, we demonstrated that down-regulation of *KIF20A*, thereby disruption of the normal process of cytokinesis and induction of mitotic arrest, is among mechanisms of action of fisetin on cancer cells.

Our results showed that fisetin is able to induce apoptosis in cancer cells, is in complete agreement with previous studies in human cancer cells [15,42,62,63]

It is well known that topoisomerase inhibitors, including doxorubicin and etoposide are widely used antitumor agents [64,65]. Previous results reported that fisetin is a powerful DNA topoisomerase II poison [66]. Here, we showed that fisetin inhibited the transcription of topoisomerase II A (*TOP2A*) enzyme in human HepG-2 and Suit-2 cancer cells and that could be responsible, in part, for the anti-tumor effect of fisetin on those cells.

Drug-drug interaction in cancer patients can lead to either overdosing or under-treatment resulting in unexpected and/or severe clinical consequences. Additionally, herbal medicines, which are orally administered, could affect bioavailability of co-administered drugs. In addition, several herbs can give rise to the potential of harmful interactions with targeted agents [67,68]. In our study, we aimed to test the inhibitory effects of fisetin on CYP3A4 involved in the hepatic metabolism of most drugs. Fisetin was found to exhibit a significant and dose-dependent inhibitory activity against CYP3A4 enzyme indicating a critical sign and/or alarm during development of fisetin-based antitumor strategies and/or combination remedies.

Overexpression of glutathione S-transferases (GST) and efflux pumps in cancer cells may reduce the antitumor activity of various chemotherapeutic agents. Recently, it has become evident that glutathione S-transferases are also involved in the control of apoptosis through involvement of the JNK signaling pathway [69]. Here, we reported that fisetin was able to inhibit GST in a dose-dependent manner indicating a rational for combination-based therapies with fisetin.

Our transcript profile analysis results could be used as starting point for the generation of theories on candidate genes and for a more detailed segmentation of the role of individual transcripts for the activity of fisetin in hepatic and pancreatic cancer cells. Additionally, we provide new insights into fisetin-related signaling pathways and networks that may facilitate the development of fisetin-based antitumor strategies and/or combination remedies.

## Conclusion

The present analysis clearly demonstrated that fisetin inhibited hepatic, colorectal and pancreatic cancer cellular growth and proliferation through affecting important and multiple signaling pathways involved in tumor cell growth and differentiations. The inhibitory effect of fisetin was mainly mediated through activation of *CDKN1A*, *SEMA3E*, *GADD45B* and *GADD45A* and down-regulation of *TOP2A*, *KIF20A*, *CCNB2* and *CCNB1* genes. Our results and the expression analyses data reported here could be used to as a tool for further studies for the development of fisetin-based antitumor strategies and/or combination therapies.

## References

[1] Tang S, Wu WK, Li X, Wong SH, Wong N, Chan MT, et al (2016) Stratification of Digestive Cancers with Different Pathological Features and Survival Outcomes by MicroRNA Expression. Sci Rep 6: 24466 10.1038/srep24466 27080237 PMC4832245

[2] Ferlay J, Shin HR, Bray F, Forman D, Mathers C, Parkin DM (2010) Estimates of worldwide burden of cancer in 2008: GLOBOCAN 2008. Int J Cancer 127: 2893–2917. 10.1002/ijc.25516 21351269

[3] Vatandoost N, Ghanbari J, Mojaver M, Avan A, Ghayour-Mobarhan M, Nedaeinia R, et al (2016) Early detection of colorectal cancer: from conventional methods to novel biomarkers. J Cancer Res Clin Oncol 142: 341–351. 10.1007/s00432-015-1928-z 25687380 PMC11819464

[4] Youns M, Fathy GM (2013) Upregulation of extrinsic apoptotic pathway in curcumin-mediated antiproliferative effect on human pancreatic carcinogenesis. J Cell Biochem 114: 2654–2665. 10.1002/jcb.24612 23794119

[5] Vedeld HM, Andresen K, Eilertsen IA, Nesbakken A, Seruca R, Gladhaug IP, et al (2015) The novel colorectal cancer biomarkers CDO1, ZSCAN18 and ZNF331 are frequently methylated across gastrointestinal cancers. Int J Cancer 136: 844–853. 10.1002/ijc.29039 24948044 PMC4277335

[6] Khan N, Syed DN, Ahmad N, Mukhtar H (2013) Fisetin: a dietary antioxidant for health promotion. Antioxid Redox Signal 19: 151–162. 10.1089/ars.2012.4901 23121441 PMC3689181

[7] Chung MY, Lim TG, Lee KW (2013) Molecular mechanisms of chemopreventive phytochemicals against gastroenterological cancer development. World J Gastroenterol 19: 984–993. 10.3748/wjg.v19.i7.984 23467658 PMC3582010

[8] Lee KW, Bode AM, Dong Z (2011) Molecular targets of phytochemicals for cancer prevention. Nat Rev Cancer 11: 211–218. 10.1038/nrc3017 21326325

[9] Arai Y, Watanabe S, Kimira M, Shimoi K, Mochizuki R, Kinae N (2000) Dietary intakes of flavonols, flavones and isoflavones by Japanese women and the inverse correlation between quercetin intake and plasma LDL cholesterol concentration. J Nutr 130: 2243–2250. 10958819 10.1093/jn/130.9.2243

[10] Haddad AQ, Venkateswaran V, Viswanathan L, Teahan SJ, Fleshner NE, Klotz LH (2006) Novel antiproliferative flavonoids induce cell cycle arrest in human prostate cancer cell lines. Prostate Cancer Prostatic Dis 9: 68–76. 10.1038/sj.pcan.4500845 16314891

[11] Pal HC, Sharma S, Elmets CA, Athar M, Afaq F (2013) Fisetin inhibits growth, induces G(2) /M arrest and apoptosis of human epidermoid carcinoma A431 cells: role of mitochondrial membrane potential disruption and consequent caspases activation. Exp Dermatol 22: 470–475. 10.1111/exd.12181 23800058 PMC3725735

[12] Maher P, Akaishi T, Abe K (2006) Flavonoid fisetin promotes ERK-dependent long-term potentiation and enhances memory. Proc Natl Acad Sci U S A 103: 16568–16573. 10.1073/pnas.0607822103 17050681 PMC1637622

[13] Chen Y, Wu Q, Song L, He T, Li Y, Li L, et al (2015) Polymeric micelles encapsulating fisetin improve the therapeutic effect in colon cancer. ACS Appl Mater Interfaces 7: 534–542. 10.1021/am5066893 25495760

[14] Chen CM, Hsieh YH, Hwang JM, Jan HJ, Hsieh SC, Lin SH, et al (2015) Fisetin suppresses ADAM9 expression and inhibits invasion of glioma cancer cells through increased phosphorylation of ERK1/2. Tumour Biol 36: 3407–3415. 10.1007/s13277-014-2975-9 25527158

[15] Kang KA, Piao MJ, Hyun JW (2015) Fisetin induces apoptosis in human nonsmall lung cancer cells via a mitochondria-mediated pathway. In Vitro Cell Dev Biol Anim 51: 300–309. 10.1007/s11626-014-9830-6 25381036

[16] Li R, Zhao Y, Chen J, Shao S, Zhang X (2014) Fisetin inhibits migration, invasion and epithelial-mesenchymal transition of LMP1-positive nasopharyngeal carcinoma cells. Mol Med Rep 9: 413–418. 10.3892/mmr.2013.1836 24297333

[17] Khan MI, Adhami VM, Lall RK, Sechi M, Joshi DC, Haidar OM, et al (2014) YB-1 expression promotes epithelial-to-mesenchymal transition in prostate cancer that is inhibited by a small molecule fisetin. Oncotarget 5: 2462–2474. 10.18632/oncotarget.1790 24770864 PMC4058019

[18] Li J, Qu W, Cheng Y, Sun Y, Jiang Y, Zou T, et al (2014) The inhibitory effect of intravesical fisetin against bladder cancer by induction of p53 and down-regulation of NF-kappa B pathways in a rat bladder carcinogenesis model. Basic Clin Pharmacol Toxicol 115: 321–329. 10.1111/bcpt.12229 24646039

[19] Chou RH, Hsieh SC, Yu YL, Huang MH, Huang YC, Hsieh YH (2013) Fisetin inhibits migration and invasion of human cervical cancer cells by down-regulating urokinase plasminogen activator expression through suppressing the p38 MAPK-dependent NF-kappaB signaling pathway. PLoS One 8: e71983 10.1371/journal.pone.0071983 23940799 PMC3733924

[20] Syed DN, Afaq F, Maddodi N, Johnson JJ, Sarfaraz S, Ahmad A, et al (2011) Inhibition of human melanoma cell growth by the dietary flavonoid fisetin is associated with disruption of Wnt/beta-catenin signaling and decreased Mitf levels. J Invest Dermatol 131: 1291–1299. 10.1038/jid.2011.6 21346776 PMC3166244

[21] Seo SH, Jeong GS (2015) Fisetin inhibits TNF-alpha-induced inflammatory action and hydrogen peroxide-induced oxidative damage in human keratinocyte HaCaT cells through PI3K/AKT/Nrf-2-mediated heme oxygenase-1 expression. Int Immunopharmacol 29: 246–253. 10.1016/j.intimp.2015.11.014 26590114

[22] Zhuo W, Zhang L, Zhu Y, Zhu B, Chen Z (2015) Fisetin, a dietary bioflavonoid, reverses acquired Cisplatin-resistance of lung adenocarcinoma cells through MAPK/Survivin/Caspase pathway. Am J Transl Res 7: 2045–2052. 26692948 PMC4656781

[23] Smith ML, Murphy K, Doucette CD, Greenshields AL, Hoskin DW (2016) The Dietary Flavonoid Fisetin Causes Cell Cycle Arrest, Caspase-Dependent Apoptosis, and Enhanced Cytotoxicity of Chemotherapeutic Drugs in Triple-Negative Breast Cancer Cells. J Cell Biochem 117: 1913–1925. 10.1002/jcb.25490 26755433

[24] Youns M, Efferth T, Reichling J, Fellenberg K, Bauer A, Hoheisel JD (2009) Gene expression profiling identifies novel key players involved in the cytotoxic effect of Artesunate on pancreatic cancer cells. Biochem Pharmacol 78: 273–283. 10.1016/j.bcp.2009.04.014 19393226

[25] Nasr T, Bondock S, Youns M (2014) Anticancer activity of new coumarin substituted hydrazide-hydrazone derivatives. Eur J Med Chem 76: 539–548. 10.1016/j.ejmech.2014.02.026 24607878

[26] Alhamdani MS, Youns M, Buchholz M, Gress TM, Beckers MC, Marechal D, et al (2012) Immunoassay-based proteome profiling of 24 pancreatic cancer cell lines. J Proteomics 75: 3747–3759. 10.1016/j.jprot.2012.04.042 22579748

[27] Torres Salazar A, Hoheisel J, Youns M, Wink M (2011) Anti-inflammatory and anti-cancer activities of essential oils and their biological constituents. Int J Clin Pharmacol Ther 49: 93–95. 21176743

[28] Ahmed MF, Youns M (2013) Synthesis and biological evaluation of a novel series of 6,8-dibromo-4(3H)quinazolinone derivatives as anticancer agents. Arch Pharm (Weinheim) 346: 610–617. 23873839 10.1002/ardp.201300158

[29] Youns M, Efferth T, Hoheisel JD (2011) Transcript profiling identifies novel key players mediating the growth inhibitory effect of NS-398 on human pancreatic cancer cells. Eur J Pharmacol 650: 170–177. 10.1016/j.ejphar.2010.10.026 20969859

[30] Mulyaningsih S, Youns M, El-Readi MZ, Ashour ML, Nibret E, Sporer F, et al (2010) Biological activity of the essential oil of Kadsura longipedunculata (Schisandraceae) and its major components. J Pharm Pharmacol 62: 1037–1044. 10.1111/j.2042-7158.2010.01119.x 20663038

[31] de Souza Rocha Simonini P, Breiling A, Gupta N, Malekpour M, Youns M, Omranipour R, et al (2010) Epigenetically deregulated microRNA-375 is involved in a positive feedback loop with estrogen receptor alpha in breast cancer cells. Cancer Res 70: 9175–9184. 10.1158/0008-5472.CAN-10-1318 20978187

[32] Cali JJ, Ma D, Sobol M, Simpson DJ, Frackman S, Good TD, et al (2006) Luminogenic cytochrome P450 assays. Expert Opin Drug Metab Toxicol 2: 629–645. 10.1517/17425255.2.4.629 16859410

[33] Habig WH, Pabst MJ, Jakoby WB (1974) Glutathione S-transferases. The first enzymatic step in mercapturic acid formation. J Biol Chem 249: 7130–7139. 4436300

[34] Igney FH, Krammer PH (2002) Death and anti-death: tumour resistance to apoptosis. Nat Rev Cancer 2: 277–288. 10.1038/nrc776 12001989

[35] Park W, Amin AR, Chen ZG, Shin DM (2013) New perspectives of curcumin in cancer prevention. Cancer Prev Res (Phila) 6: 387–400. 10.1158/1940-6207.CAPR-12-0410 PMC3693758 23466484

[36] Youns M, Hoheisel JD, Efferth T (2010) Toxicogenomics for the prediction of toxicity related to herbs from traditional Chinese medicine. Planta Med 76: 2019–2025. 10.1055/s-0030-1250432 20957595

[37] Youns M, Efferth T, Hoheisel JD (2009) Microarray analysis of gene expression in medicinal plant research. Drug Discov Ther 3: 200–207. 22495629

[38] Piao MJ, Kim KC, Chae S, Keum YS, Kim HS, Hyun JW (2013) Protective Effect of Fisetin (3,7,3',4'-Tetrahydroxyflavone) against gamma-Irradiation-Induced Oxidative Stress and Cell Damage. Biomol Ther (Seoul) 21: 210–215. 24265866 10.4062/biomolther.2013.017 PMC3830119

[39] Kang KA, Piao MJ, Kim KC, Cha JW, Zheng J, Yao CW, et al (2014) Fisetin attenuates hydrogen peroxide-induced cell damage by scavenging reactive oxygen species and activating protective functions of cellular glutathione system. In Vitro Cell Dev Biol Anim 50: 66–74. 10.1007/s11626-013-9681-6 23982916

[40] Yang PM, Tseng HH, Peng CW, Chen WS, Chiu SJ (2012) Dietary flavonoid fisetin targets caspase-3-deficient human breast cancer MCF-7 cells by induction of caspase-7-associated apoptosis and inhibition of autophagy. Int J Oncol 40: 469–478. 10.3892/ijo.2011.1203 21922137

[41] Li J, Cheng Y, Qu W, Sun Y, Wang Z, Wang H, et al (2011) Fisetin, a dietary flavonoid, induces cell cycle arrest and apoptosis through activation of p53 and inhibition of NF-kappa B pathways in bladder cancer cells. Basic Clin Pharmacol Toxicol 108: 84–93. 10.1111/j.1742-7843.2010.00613.x 21054790

[42] Suh Y, Afaq F, Johnson JJ, Mukhtar H (2009) A plant flavonoid fisetin induces apoptosis in colon cancer cells by inhibition of COX2 and Wnt/EGFR/NF-kappaB-signaling pathways. Carcinogenesis 30: 300–307. 10.1093/carcin/bgn269 19037088 PMC2722149

[43] Khan N, Afaq F, Syed DN, Mukhtar H (2008) Fisetin, a novel dietary flavonoid, causes apoptosis and cell cycle arrest in human prostate cancer LNCaP cells. Carcinogenesis 29: 1049–1056. 10.1093/carcin/bgn078 18359761 PMC2902387

[44] Khan N, Asim M, Afaq F, Abu Zaid M, Mukhtar H (2008) A novel dietary flavonoid fisetin inhibits androgen receptor signaling and tumor growth in athymic nude mice. Cancer Res 68: 8555–8563. 10.1158/0008-5472.CAN-08-0240 18922931 PMC2954499

[45] Chen YC, Shen SC, Lee WR, Lin HY, Ko CH, Shih CM, et al (2002) Wogonin and fisetin induction of apoptosis through activation of caspase 3 cascade and alternative expression of p21 protein in hepatocellular carcinoma cells SK-HEP-1. Arch Toxicol 76: 351–359. 10.1007/s00204-002-0346-6 12107653

[46] Suh Y, Afaq F, Khan N, Johnson JJ, Khusro FH, Mukhtar H (2010) Fisetin induces autophagic cell death through suppression of mTOR signaling pathway in prostate cancer cells. Carcinogenesis 31: 1424–1433. 10.1093/carcin/bgq115 20530556 PMC2915634

[47] Ramachandran C, Rodriguez S, Ramachandran R, Raveendran Nair PK, Fonseca H, Khatib Z, et al (2005) Expression profiles of apoptotic genes induced by curcumin in human breast cancer and mammary epithelial cell lines. Anticancer Res 25: 3293–3302. 16101141

[48] Chen H, Xie GH, Wang WW, Yuan XL, Xing WM, Liu HJ, et al (2015) Epigenetically downregulated Semaphorin 3E contributes to gastric cancer. Oncotarget 6: 20449–20465. 10.18632/oncotarget.3936 26036259 PMC4653017

[49] Casazza A, Finisguerra V, Capparuccia L, Camperi A, Swiercz JM, Rizzolio S, et al (2010) Sema3E-Plexin D1 signaling drives human cancer cell invasiveness and metastatic spreading in mice. J Clin Invest 120: 2684–2698. 10.1172/JCI42118 20664171 PMC2912191

[50] Biankin AV, Waddell N, Kassahn KS, Gingras MC, Muthuswamy LB, Johns AL, et al (2012) Pancreatic cancer genomes reveal aberrations in axon guidance pathway genes. Nature 491: 399–405. 10.1038/nature11547 23103869 PMC3530898

[51] Tamura RE, de Vasconcellos JF, Sarkar D, Libermann TA, Fisher PB, Zerbini LF (2012) GADD45 proteins: central players in tumorigenesis. Curr Mol Med 12: 634–651. 22515981 10.2174/156652412800619978 PMC3797964

[52] Liebermann DA, Hoffman B (2007) Gadd45 in the response of hematopoietic cells to genotoxic stress. Blood Cells Mol Dis 39: 329–335. 10.1016/j.bcmd.2007.06.006 17659913 PMC3268059

[53] Sajadian SO, Tripura C, Samani FS, Ruoss M, Dooley S, Baharvand H, et al (2016) Vitamin C enhances epigenetic modifications induced by 5-azacytidine and cell cycle arrest in the hepatocellular carcinoma cell lines HLE and Huh7. Clin Epigenetics 8: 46 10.1186/s13148-016-0213-6 27134688 PMC4851801

[54] Hoffman B, Liebermann DA (2007) Role of gadd45 in myeloid cells in response to hematopoietic stress. Blood Cells Mol Dis 39: 344–347. 10.1016/j.bcmd.2007.06.011 17686638 PMC2684334

[55] Zerbini LF, Libermann TA (2005) GADD45 deregulation in cancer: frequently methylated tumor suppressors and potential therapeutic targets. Clin Cancer Res 11: 6409–6413. 10.1158/1078-0432.CCR-05-1475 16166414

[56] Qiu W, Zhou B, Zou H, Liu X, Chu PG, Lopez R, et al (2004) Hypermethylation of growth arrest DNA damage-inducible gene 45 beta promoter in human hepatocellular carcinoma. Am J Pathol 165: 1689–1699. 15509538 10.1016/s0002-9440(10)63425-6 PMC1618679

[57] Shi Q, Wang W, Jia Z, Chen P, Ma K, Zhou C (2016) ISL1, a novel regulator of CCNB1, CCNB2 and c-MYC genes, promotes gastric cancer cell proliferation and tumor growth. Oncotarget. 10.18632/oncotarget.9269 PMC5095015 27183908

[58] Daire V, Pous C (2011) Kinesins and protein kinases: key players in the regulation of microtubule dynamics and organization. Arch Biochem Biophys 510: 83–92. 10.1016/j.abb.2011.02.012 21345331

[59] Yan GR, Zou FY, Dang BL, Zhang Y, Yu G, Liu X, et al (2012) Genistein-induced mitotic arrest of gastric cancer cells by downregulating KIF20A, a proteomics study. Proteomics 12: 2391–2399. 10.1002/pmic.201100652 22887948

[60] Hill E, Clarke M, Barr FA (2000) The Rab6-binding kinesin, Rab6-KIFL, is required for cytokinesis. EMBO J 19: 5711–5719. 10.1093/emboj/19.21.5711 11060022 PMC305783

[61] Taniuchi K, Nakagawa H, Nakamura T, Eguchi H, Ohigashi H, Ishikawa O, et al (2005) Down-regulation of RAB6KIFL/KIF20A, a kinesin involved with membrane trafficking of discs large homologue 5, can attenuate growth of pancreatic cancer cell. Cancer Res 65: 105–112. 15665285

[62] Kim JA, Lee S, Kim DE, Kim M, Kwon BM, Han DC (2015) Fisetin, a dietary flavonoid, induces apoptosis of cancer cells by inhibiting HSF1 activity through blocking its binding to the hsp70 promoter. Carcinogenesis 36: 696–706. 10.1093/carcin/bgv045 25840992

[63] Ying TH, Yang SF, Tsai SJ, Hsieh SC, Huang YC, Bau DT, et al (2012) Fisetin induces apoptosis in human cervical cancer HeLa cells through ERK1/2-mediated activation of caspase-8-/caspase-3-dependent pathway. Arch Toxicol 86: 263–273. 10.1007/s00204-011-0754-6 21964635

[64] Schmidt BH, Osheroff N, Berger JM (2012) Structure of a topoisomerase II-DNA-nucleotide complex reveals a new control mechanism for ATPase activity. Nat Struct Mol Biol 19: 1147–1154. 10.1038/nsmb.2388 23022727 PMC3492516

[65] Berger JM, Gamblin SJ, Harrison SC, Wang JC (1996) Structure and mechanism of DNA topoisomerase II. Nature 379: 225–232. 10.1038/379225a0 8538787

[66] Olaharski AJ, Mondrala ST, Eastmond DA (2005) Chromosomal malsegregation and micronucleus induction in vitro by the DNA topoisomerase II inhibitor fisetin. Mutat Res 582: 79–86. 10.1016/j.mrgentox.2005.01.002 15781213

[67] Jung H, Lee S (2014) Inhibition of Human Cytochrome P450 Enzymes by Allergen Removed Rhus verniciflua Stoke Standardized Extract and Constituents. Evid Based Complement Alternat Med 2014: 150351 10.1155/2014/150351 25061471 PMC4100265

[68] Hwang SW, Han HS, Lim KY, Han JY (2008) Drug interaction between complementary herbal medicines and gefitinib. J Thorac Oncol 3: 942–943. 10.1097/JTO.0b013e3181803f1e 18670318

[69] Sau A, Pellizzari Tregno F, Valentino F, Federici G, Caccuri AM (2010) Glutathione transferases and development of new principles to overcome drug resistance. Arch Biochem Biophys 500: 116–122. 10.1016/j.abb.2010.05.012 20494652

